# Editorial: Women in science: aging and public health 2022

**DOI:** 10.3389/fpubh.2023.1226240

**Published:** 2023-06-13

**Authors:** Colette J. Browning, Marcia G. Ory

**Affiliations:** ^1^Health Innovation and Transformation Centre, Federation University Australia, Ballarat, VIC, Australia; ^2^Research School of Population Health, Australian National University, Canberra, ACT, Australia; ^3^Center for Community Health and Aging, Texas A&M University, College Station, TX, United States

**Keywords:** health and aging, lifestyle behaviors, social isolation, age-related diseases, aged care

## Introduction

As noted in our inaugural *Women in science: aging and public health 2021* Research Topic, globally there is a recognition that women are under-represented both as scientists and as participants in health research but have much to contribute to advancing public health knowledge and practice (1).

This Year's Research Topic includes 22 papers led by senior and early career women from across the world covering a range of geographies, topic areas, methodologies, and data collection sites. We highlight research studies conducted in Asia, Australia, Europe, the United Kingdom, and the United States of America, as well as reviews which are not country specific. This year's papers explore timely topical themes such as social determinants of health including gender effects, and conditions where women experience significant disease burden (breast cancer, cardiovascular disease, falls, and osteoarthritis). Many of the papers report findings from large-scale longitudinal studies on aging including the Chinese Longitudinal Healthy Longevity Survey (CLHLS), the China Health and Retirement Study (CHARLS), the China Health and Nutrition Survey (CHNS), the China Family Panel Study (CFPS), and the English Longitudinal Study of Aging (ELSA). While others are smaller, focussed studies in under-researched or difficult to reach populations. The papers in this Research Topic include systematic reviews, and studies that identify risk factors for disease and disability that can enhance the effectiveness of public health and aging interventions. Specific areas covered include functional and health capacity, physical activity and sedentary behavior, social isolation and social support, age -related diseases, and health and aged care. The career trajectories of three women scientists who have made salient contributions to aging and public health are envisioned through their colleagues. These three thought leaders in aging and public health research (Adepoju; Pachana; Vollmer Dahlke) represent women scientists who have established stellar careers while mentoring others. A common theme is their passion for their research and recognition of the importance of community.

## Functional and health capacity in various roles

At a population level, as people age, functional capacity and health status declines and disability increases. However, decline is not inevitable for all groups nor individuals, and appropriate interventions that address personal as well as social and environmental impacts can mitigate these trajectories (2, 3).

While disability is often associated with depression in older people, Tian et al. examined the potential moderating effect of social support on that relationship. Using data from the CLHLS of adults aged 65 years and over, 26.8% of the sample exhibited depressive symptoms, 31% showed limited activities of daily living and social support reduced the impact of disability on depressive symptoms. As with other research stressing the nuances of different types and amounts of social support (4), the effect of social support measured according to who provided the support potentially reflects the importance of the closeness of the source of support rather than simply frequency of contact. This finding has implications for recent COVID-19 social interaction restrictions.

Examining trajectories of aging outcomes using longitudinal methods has increased over the last decade. Such approaches provide stronger evidence about the predictors of healthy aging and the opportunities for interventions to address modifiable risk factors. Using data from the CLHLS, Zhao et al. identified four trajectories of physical functioning over 16 years as measured by ADL and IADL in adults aged 64 years and over: stable (35.4%), slow decline (33.0%), rapid decline (23.5%), poor function, and moderate decline (8.1%). Predictors of the trajectories included older age and male sex as well as potential modifiable risk factors such as poorer vision status, more chronic illnesses, poorer cognitive function, lack of exercise and decreased leisure activity, and depressive symptoms. It is important to address potentially modifiable risk factors associated with increased rates of decline in physical functioning.

Population aging has resulted in more pressure for older people to continue in the workforce beyond “normal” retirement age. In China, the retirement age for men is between 55 and 60 years and for women between 50 and 55 years depending upon occupation. “Early” legislated retirement in China and the impact of COVID-19 on early retirement in other countries such as the US have put pressure on labor availability and pension systems. Cheng et al. propose that there is an untapped work capacity in older Chinese women. Using data from CHARLS they examined the health capacity of Chinese women aged 45–74 years to support a delay in retirement. They concluded that urban women, particularly those with higher education, have higher “excess” capacity to work than less healthy, under-educated rural women who in contrast show higher labor participation rates. The reluctance of urban women to continue working may be related to caring for grandchildren, and incentives to keep women in the workforce need to recognize the disproportionate burden that women face as carers (5).

In many countries continuing to drive is seen as a marker of independence and facilitator of social and work connections. One study in this Research Topic (Mielenz et al.) investigated driving cessation prevalence and the relationships with self-care health behaviors. This US study of active drivers aged 65–79 years found that driving cessation prevalence at a 6-year follow up was low (0.63 per 100 person years). However, the ability to participate in and derive satisfaction with social roles and activities was protective for driving cessation. The relationship between loss of community engagement and loss of independence through driving cessation reflects unintended consequences that may be experienced by older adults in response to restrictive emergency management guidelines.

## Physical activity/sedentary behavior

It is well-established that physical activity is a protective factor for health and wellbeing as we age, and sedentary behavior is a risk factor for many chronic conditions (6, 7). The papers in this section focussed on physical activity and/or sedentary behavior in different countries and cultures, including literature reviews linking physical activity/sedentary behaviors to key geriatric conditions, observational studies examining prevalence rates and interactions, and small intervention efforts.

A major public health question is the type and amount of physical activity needed to show positive health outcomes. In a systematic review, Alzar-Teruel et al. examine the impact of different intensity levels on key healthy aging indicators such as body composition and muscle strength. While they find positive outcomes associated with high-intensity interval training, they could not demonstrate a benefit over moderate-intensity continuous training for physical functioning. More research is needed to better explicate what exercise regimens are best in different populations and settings, and guide public health recommendations.

Baseline data from the European (Austria, France, Germany, Portugal, and Switzerland) Do-Health clinical trial (Mattle et al.) was used to investigate the prevalence of physical activity (PA) and sedentary behavior (SB) in community dwelling adults aged 70 years and over. While almost two-thirds (62.2%) met physical activity recommendations, over a third (37.1%) spent at least 5.5 h per day sedentary. Physical activity prevalence but not sedentary behavior varied across the country sites, and older age, female sex, and higher BMI groups were less likely to meet the PA recommendations. These initial variations in prevalence warrant a consideration of individualized public health interventions for those at greater risk.

Luo et al. investigated the mediating role of physical activity on the role of income inequality, as measured by different types of health insurance, in life satisfaction. The data source was older participants aged 60 years and over from the last 4 data collection waves (2006, 2009, 2011, 2015) of the CHNS. In China people have access to different types of health insurance depending on their geographical location (urban vs. rural) and employment status. Having any type of health insurance and physical activity were both positively associated with life satisfaction, and physical activity mediated the impact of health insurance on life satisfaction.

Two small studies are especially promising for guiding future public health actions for older adults as well as sedentary workers. Improving adherence to exercise programs, particularly post-hospital rehabilitation programs for older people, was the focus of an Australian video intervention (Vaz et al.) to facilitate home exercises. Functional mobility, gait speed, balance and physical activity participation improved at follow-up. Tailored self-modeled videos and other digital health modalities show promise in reaching those in home settings where continued exercise adherence is important for recovery process.

The impact of sedentary behavior on metabolic health has been well-established (8). Silva et al. investigated the effects of a 16-week exercise program on middle aged sedentary workers during the 2020 COVID-19 lockdown in Portugal. The intervention group had reduced waist and hip measures and fasting glucose and lipid profiles remained stable in the intervention group. The intervention group showed less perceived stress and better quality of life. Adopting and adhering to an exercise program in middle age has the potential to favorably impact healthy aging.

## Social isolation/social support

Social isolation and the impacts on older people, especially during the height of the COVID-19 pandemic, have received considerable attention in the recent research literature (9). Several studies have found that vulnerable and disadvantaged groups had worst outcomes during the pandemic (10–12).

A French study (Silberzan et al.) surveying adults aged 65 year and over during and after the COVID-19 lockdown in France found that women were more likely to be socially isolated than men. Older age, perceived financial difficulties, and belonging to an ethnic minority were also associated with social isolation. Identification of such health inequities can inform preventive policies aimed at helping the most socially isolated stay socially connected and informed about the latest health information.

Shen et al. using data from the CFPS investigated the impact of formal social support and informal social support on quality of life in China. Different patterns of impacts on quality of life were evident depending on the quality of life measure and gender status. While both formal and informal social support enhanced older adult's quality of life, informal support had the greatest impact, consistent with findings by Tian on the importance of close informal relationships.

## Age-related diseases

Cancer, cardiovascular disease, and suicide are increasingly more prevalent in later life and warrant continued attention. Deaths from cardiovascular disease following chemotherapy for breast cancer are a significant issue for breast cancer survivors. Huang et al. constructed a nomogram from the US SEER cancer database, consisting of older female patients aged 65 years and over who underwent chemotherapy between 2010 and 2015 to predict long term survival. The nomogram, based on six factors (age, race, tumor stage, surgery, and radiotherapy), was able to classify patients at different risk levels to assist with targeted management. This is an important tool in identifying breast cancer patients who will need further management to address heart disease risk.

Knee osteoarthritis (OA) is a significant contributor to disability as we age, impacting mobility and quality of life. Obesity and socioeconomic position are risk factors in OA. To study the longitudinal relationships in England, Witkam et al. used data from nine waves of ELSA. Lower socioeconomic position increased the chances of functional limitations. Yet, participants with lower income, wealth and higher deprivation were less likely to have joint replacements. These data indicate that even in a health system where medical treatment is free, unmet need is related to health inequality.

Suicide risk is an under-developed area in older populations often related to stigma, ageist attitudes and simplistic understandings of suicide prevention in older people (13). An Australian survey (Klein et al.) on suicidality in older people aged 65 years and over found that suicidality was associated with dissatisfaction with social interaction and community engagement. While social relationships can buffer the negative effects of aging and has potential as a preventive measure in suicide prevention, addressing broader social determinants of suicide (14) such as poverty, elder abuse and discrimination are important in preventing suicide in old age.

## Health and aged care

The WHO Decade of Healthy Aging highlights the importance of integrated care and high quality long-term care (2). Polypharmacy where older adults are prescribed multiple medicines to treat different conditions can have adverse effects on health, such as falls, overdoses and cognitive problems, due to medication interactions (15). A systematic review (Oktora et al.) of patients' attitudes to deprescribing found that in low-middle income countries such as Nepal and Malaysia < 70% of patients were willing to stop medications compared to patients in high-income countries such as Australia, USA and Europe where >85% of patients were willing to stop medications. Future research calls for understanding how the health care system and setting factors interact with sociodemographic factors and cultural predispositions to influence deprescribing behaviors.

Polypharmacy also increases the risk of poor medication adherence, particularly in older patients living with dementia. A study (Muñoz-Contreras et al.) located in Spain found that caregivers improved medication adherence for care recipients living with Alzheimer's disease. Female sex and first-degree relative status of the caregiver were associated with greater adherence. Training caregivers in disease management and medication adherence can help support optimum treatment for their underlying chronic illnesses.

There is a global focus on research in residential aged care settings (nursing homes) and particularly with patients living with dementia and their carers. Aging in place promotes supporting people to live in their own homes with social and health care support. However, little is known about this significant group of older people in terms of their experiences and service needs. An Australian study (Hobden et al.) of older people receiving care services in their own home investigated their experiences of patient-centered care from health professionals. Many participants responded that they had experienced many of the elements of patient centered care. However, only just over half of participants were involved in goal setting or involvement in treatment decisions. The incomplete delivery of patient centered care to older people is linked to deficiencies in the training and accreditation of health care professionals who work with older people (16).

Older people, especially those living in nursing homes were particularly vulnerable during the COVID-19 pandemic. Using health and demographic data from a Spanish cohort undergoing COVID-19 testing between 2020 and 2021, Aguilar-Palacio et al. found that 38.3% of COVID-19 confirmed patients aged 65 years and over were hospitalized. Risk of hospitalization was higher in men, older people, those with a diagnosed chronic illness and those on higher pensions. The death rate within 90 days of COVID-19 diagnosis was 31.5% and the risk of death was higher in men and older patients. Knowing patient specific risk factors will be valuable for preparing for future health threats for the most vulnerable long-term care populations.

## Conclusion

While there were many more articles to highlight in this year's 2022 collection, we still lament the relative lack of research that is theory driven and methodologically rigorous asking critical questions about women's issues or gender differences in lifestyle behaviors, access to health care and social service supports, activity friendly communities, or disease/disability states. A final article in the Research Topic emphasizes this concern. While the role of technology in supporting aging well has been highlighted elsewhere in this journal (17), Heine and Feldman review the role of technology in the quality of life of older women. Their focus was on the context of COVID-19 where many became socially isolated and needed to rely on technology for social interactions. They concluded that gender specific models were lacking in the literature and highlighted the need to consider dignity, ageism, autonomy and privacy in the design and implementation of technologies for older women. We would broaden this sentiment to highlight all these factors in aging and public health research.

On a positive note, this Research Topic shines a light on contributions that women scientists are making to further our knowledge about functional and health capacity, lifestyle behaviors associated with health risks, social isolation and social supports as major risk or protective factors, age related diseases, and health and aged care. We hope that the worst of the COVID-19 world-wide pandemic is behind us, but that we carry forth lessons learned about Research Topics of interest and the conduct of research to prepare us for future research and practice challenges that established and emerging women in science can address effectively.

## Author contributions

CB and MO conceptualized the editorial. CB led the writing of the editorial. MO provided expert feedback on the editorial. All authors contributed to the article and approved the submitted version.

## References

[1] Browning C Ory MG Pei X. Editorial: Women in science: Aging and public health 2021. Front Public Health. (2022) 10:895113. 10.3389/fpubh.2022.895113 35712317 PMC9195419

[2] World Health Organization. Decade of Healthy Ageing. Geneva: World Health Organization (2020).

[3] World Health Organization. World Report on Ageing and Health. Geneva: World Health Organization. (2015). Available online at: http//www.who.int/ageing/publications/world-report-2015-launch/en/ (accessed May 2, 2023).

[4] Benca-Bachman CE Najera DD Whitfield KE Taylor JL Thorpe RJJr Palmer RHC. Quality and quantity of social support show differential associations with stress and depression in African Americans. Am J Geriatr Psychiatry. (2020) 28:597–605. 10.1016/j.jagp.2020.02.004 32165073 PMC7246182

[5] Ferrant G Pesando LM Nowacka K. Unpaid Care Work: The Missing Link in the Analysis of Gender Gaps in Labour Outcomes. (2014). Available online at: https://www.oecd.org/dev/development-gender/Unpaid_care_work.pdf (accessed May 10, 2023).

[6] Chastin S Gardiner PA Harvey JA Leask CF Jerez-Roig J Rosenberg D. Interventions for reducing sedentary behaviour in community-dwelling older adults. Cochr Database Systemat Rev. (2021) 2021:CD012784. 10.1002/14651858.CD012784.pub2 34169503 PMC8225503

[7] Cochrane Special Collections. Physical Activity for Healthy Ageing. (2021). Available online at: https://www.cochranelibrary.com/collections/doi/SC000048/full (accessed May 2, 2023).

[8] Edwardson CL Gorely T Davies MJ Gray LJ Khunti K Wilmot EG. Association of sedentary behaviour with metabolic syndrome: A meta-analysis. PLoS ONE. (2012) 7:e34916. 10.1371/journal.pone.0034916 22514690 PMC3325927

[9] Nicklett EJ Ory MG Dwolatzky T. COVID-19, Aging, and Public Health. Lausanne: Frontiers Media SA (2022). 10.3389/fpubh.2022.924591 PMC9234639 35769770

[10] Adepoju O Chae M Woodard L Smith KL Han D Howard DL. Correlates of social isolation among community-dwelling older adults during the COVID-19 pandemic. Front Public Health. (2021) 9:702965. 10.3389/fpubh.2021.702965 34956998 PMC8702646

[11] Athavale P Kumar V Clark J Mondal S Sur S. Differential impact of COVID-19 risk factors on ethnicities in the United States. Front Public Health. (2021) 9:743003. 10.3389/fpubh.2021.743003 34938701 PMC8687082

[12] O'Sullivan D Rahamathulla M Pawar M. The impact and implications of COVID-19: An Australian perspective. Int J Commun Soc Dev. (2020) 2:134–51. 10.1177/2516602620937922

[13] De Leo D. Late-life suicide in an aging world. Nat Aging. (2022) 2:7–12. 10.1038/s43587-021-00160-1 37118360

[14] Shand F Yip D Tye M Darwin L. The Impacts of Social Determinants on Suicide How Policy Settings Can Help. (2020). Available online at: https://www.blackdoginstitute.org.au/wp-content/uploads/2020/09/What-Can-Be-Done-To-Decrease-Suicide_Chapter-2-Social-Determinants.pdf (accessed April 20, 2023).

[15] National Institute on Aging. The Dangers of Polypharmacy and the Case for Deprescribing in Older Adults. (2021). Available online at: https://www.nia.nih.gov/news/dangers-polypharmacy-and-case-deprescribing-older-adults (accessed May 11, 2023).

[16] Evashwick C. Building the workforce to care for the aged: Can accreditation contribute? Front. Public Health. (2022) 10:1062469. 10.3389/fpubh.2022.1062469 36438263 PMC9685986

[17] Levkoff SE Ory MG Chen H Kort H. Technological Innovations to Address Social Isolation and Loneliness in Older Adults. Lausanne: Frontiers Media SA. (2022). 10.3389/fpubh.2023.1139266 PMC9940659 36815157

